# Oxiapoptophagy in Age-Related Diseases. Comment on Ouyang et al. 7-Ketocholesterol Induces Oxiapoptophagy and Inhibits Osteogenic Differentiation in MC3T3-E1 Cells. *Cells* 2022, *11*, 2882

**DOI:** 10.3390/cells11223612

**Published:** 2022-11-15

**Authors:** Imen Ghzaiel, Thomas Nury, Amira Zarrouk, Anne Vejux, Gérard Lizard

**Affiliations:** 1 Team ‘Biochemistry of the Peroxisome, Inflammation and Lipid Metabolism’ EA7270/Inserm, University Bourgogne Franche-Comté, 21000 Dijon, France; 2 Lab-NAFS ‘Nutrition—Functional Food & Vascular Health’, Faculty of Medicine, University of Monastir, LR12ES05, Monastir 5000, Tunisia; 3 Faculty of Sciences of Tunis, University Tunis-El Manar, Tunis 2092, Tunisia; 4 Faculty of Medicine, University of Sousse, Sousse 4000, Tunisia

Due to the increase in life span and life expectancy, which can, however, be more or less pronounced depending on the economic, social and cultural context [1,2], age-related diseases (cardiovascular, neurodegenerative and ocular diseases, osteoporosis and sarcopenia) are becoming more and more frequent, requiring adapted care structures and leading to an increase in individual and collective health expenses.

Therefore, a better understanding of the intrinsic and extrinsic factors that promote the transition from physiological to pathological aging is essential to prevent age-related diseases in order to make the best use of the additional time that longevity brings [3].

The results presented by Jing Ouyang et al. in “7-ketocholesterol induces oxiapoptophagy and inhibits osteogenic differentiation in MC3T3-E1” [4] are important in the context of the pathophysiology of age-related diseases mainly at three levels:

- they reinforce the hypothesis that 7-ketocholesterol (7KC), essentially formed by cholesterol auto-oxidation [5], is a potential risk factor that could promote pathological aging and contribute to several age-related diseases [6,7,8] (Figure 1);
- they confirm that 7KC can induce oxiapoptophagy, originally described in 2003 [9], involving oxidative stress, apoptosis and autophagy [10]; the signaling pathways associated with this type of death are well studied [11], which makes it possible to consider identifying specific therapeutic targets to prevent this type of cell death, which could occur in several age-related diseases. In addition, the in vitro study of oxiapoptophagy has permitted the identification of cytoprotective natural and synthethic molecules as well as a mixture of molecules (especially Mediterranean oils) that counteract 7KC-induced oxiapoptophagy and that can be of therapeutic and nutraceutic interest [12,13,14];
- they provide new evidence of the potentially negative impact of 7KC in osteoporosis [15,16]; indeed, Jing Ouyang et al. show on mouse osteoblastic MCT3-E1 cells that 7KC decreases alkaline activity (ALP), used to judge osteogenic differentiation, reduces mineralization, and induces a decrease of OPN (osteopontin) and RUNX2 (Runt-2 transcription factor), two proteins involved in osteogenic differentiation [4].

It is worth noting that, at the moment, several in vitro studies have shown that natural and synthetic oxysterols act on osteogenesis by inhibiting or activating it, especially some oxysterols [17]. Deleterious effects on osteogenesis have already been observed with 7KC [16], cholestene-3β,5α,6β-triol and 27-hydroxycholesterol [18,19,20,21,22], which is an endogenous selective estrogen receptor modulator with side effects in bone. In contrast, 7α,25-hydroxycholesterol [23], 22S-hydroxycholesterol, 22R-hydroxycholesterol as well as 20S-hydroxycholesterol and some of its synthetic derivatives have bone-inducing properties [17,24]. As several oxysterols are simultaneously present in biological fluids and tissues, it would be necessary to define the oxysterol profiles in patients with osteoporosis and then to better define the activity of these oxysterols not only in vitro but also in vivo. While the use of a single oxysterol in cellular models is an essential step in specifying its biological activities and in identifying pharmacological targets and therapeutic molecules, appropriate mixtures of oxysterols (already used to address age-related or inflammatory bowel diseases) [25,26] could also be of interest to study osteoporosis in a more physiological context. Thus, to better understand the pathophysiology of osteoporosis, it seems first necessary to identify and quantify oxysterols in patients and then to address the biological activities of these molecules in appropriate cellular models. Studies on primary cultures, bone explants or organoids associated with microfluidic approaches using 7KC alone or in mixture could complement the results obtained on MC3T3-E1 and are now required.

In the context of cell death, the present study demonstrates again that oxiapoptophagy can concern different cell types from different species [10]. This type of cell death has often been described in the presence of 7KC, but also with other oxysterols (7β-hydroxycholesterol, 24S-hydroxycholesterol, 25-hydroxycholesterol, 7α,25-dihydrocholesterol, 5,6-epoxycholesterol), on human monocytic U937 cells [9], human JJN3 and U266 myeloma cells [27], bone marrow mesenchymal cells from patients with acute leukemia [28], L929 mouse fibroblast cells [29,30], and murine nerve cells (158N oligodendrocytes, microglial BV-2 cells, N2a neuronal cells) [31,32,33,34]. The study by Jing Ouyang et al. also clearly establishes an induction of oxiapoptophagy by 7KC on mouse osteoblastic MC3T3-E1 cells.

In addition, when MC3T3-E1 cells were cultured in the presence of 7KC and pre-treated with N-acetylcysteine (NAC) (a powerful antioxidant that was initially described as preventing 7KC-induced apoptosis on human U937 monocytic cells) [35], it was observed that NAC greatly reduces oxidative stress induced by 7KC and restores RedOx balance. Thus, the increases in the expression of NADPH-oxidase 4 (NOX4), the level of malonedialdehyde (MDA, a marker of lipid peroxidation) and the superoxide dismutase (SOD) activity were almost normalized. Apoptosis and autophagy were also greatly reduced. Therefore, oxidative stress plays a major role in the induction of 7KC-induced oxiapoptophagy on MC3T3-L1 cells, which is consistent with the results obtained on many other cells treated with 7KC [36]. It is important to underline that using natural or synthetic molecules, an inhibition of oxiapoptophagy has always been observed to act directly or indirectly on oxidative stress and normalize the RedOx balance [12].

Consequently, the data obtained by Jing Ouyang et al. brings additional evidence that supports the fact that 7KC could contribute (at least in part via oxiapoptophagy induction) to osteoporosis, which is a frequent age-related disease in post-menopausal women. These data constitute an additional in vitro proof of concept on the potential role of 7KC in age-related disease. There is now a need for in vivo evidence in appropriate animal models and in humans.

## Figures and Tables

**Figure 1:**
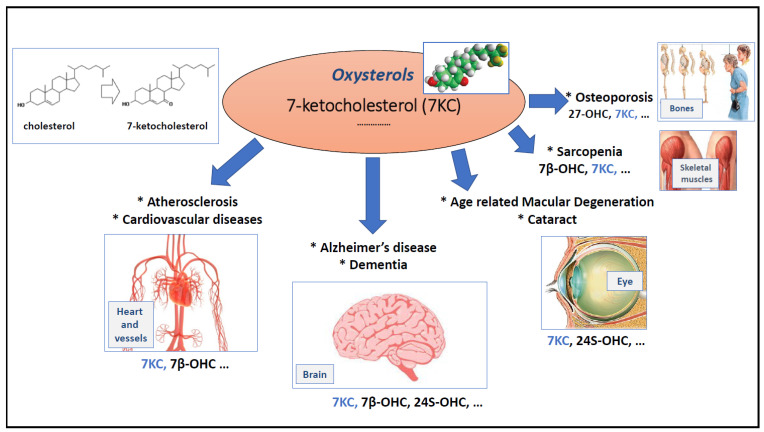
Involvement of 7-ketocholesterol (7KC) in age-related diseases [6]. The presented scheme is based on in vitro and/or in vivo proofs of concept.
